# Experimental and computational data set on adsorption of Cr (VI) from water using an activated carbon

**DOI:** 10.1016/j.dib.2020.105292

**Published:** 2020-02-15

**Authors:** Anyi Ramirez, Raúl Ocampo, Stephanie Giraldo, Erika Padilla, Elizabeth Flórez, Nancy Acelas

**Affiliations:** a Group of Materials with Impact (Mat&mpac), Department of Basic Sciences, University of Medellín, Medellín, Colombia; b Department of Chemical Sciences, Autonomous University San Luis Potosí (UASLP), San Luis Potosí, Mexico

**Keywords:** Hexavalent chromium, Adsorption, Kinetics, Isotherms, Computational simulation

## Abstract

Chromium (Cr) is a widely used metal in metallurgical and chemical industries, whose waste contaminates the surface and groundwater. Cr (VI) is toxic and produces carcinogenic effects owing to its high mobility in water and soil. In this work, computational and experimental studies from the adsorption of Cr(VI) from aqueous solutions on teak wood residues activated with ZnCl_2_ (AT) are presented. Full interpretation of data can be found in DOI:10.1016/j.jece.2020.103702 [1]. Experimental data were adjusted to Langmuir, Freundlich and Temkin isothermal models and the nonlinear and linear forms of the Pseudo-first and Pseudo-second order kinetic models. Computational data allow to understand the adsorption process of Cr(VI) on carbonaceous materials.

**Table:** Specifications Table

Subject	Environmental Science
Specific subject area	Environmental Science (General)
Type of data	Tables Figures
How data were acquired	Data were obtained by UV/VIS spectrophotometry and Density Functional Theory
Data format	Raw and analyzed
Parameters for data collection	Experimental data were collected by sampling at different contact time and changing the initial Cr (VI) concentration
Description of data collection	All experimental tests were carried out on a three-layer glass reactor placed in a constant-temperature bath at 25 °C. Reactor was stirred at 200 rpm with a turbine propeller operated by a rotor.
Data source location	Autonomous University San Luis Potosí (UASLP), San Luis Potosí, Mexico and University of Medellín, Medellín, Colombia.
Data accessibility	The raw datafiles are provided in the Data in Brief as supplementary material.
Related research article	A. Ramirez, R. Ocampo, S. Giraldo, E. Padilla, E. Flórez and N. Acelas, Removal of Cr (VI) from an aqueous solution using an activated carbon obtained from teakwood sawdust: kinetics, equilibrium, and density functional theory calculations. Journal of Environmental Chemical Engineering, Volume 8, Issue 2, April 2020, 103702. DOI:10.1016/j.jece.2020.103702

**Table:** 

**Value of the Data** • Computational data is useful to describe the interaction between Cr^3+^ and HCrO_4_^−^ on carbonaceous surfaces, and thus are essential to predict the better properties of the adsorbents (functional groups on surfaces) during experimental design of materials. • Data of isotherms and kinetics is informative to predict and model the adsorption of Cr (VI) from water. They are also useful for the academic community to complete research on anion adsorption. • Adsorption isotherms, kinetics and computational data allow to predict several important issues (adsorption capacity, surface properties and adsorption mechanism) which can advance elaboration of renewable, efficient, novel and low cost adsorbent materials for removal of Cr (VI) from water; with good potential application in the water treatment industry. • Data in this study have significance for improving water quality with the removal of Cr (VI) and others heavy metal cations using a low-cost and selective adsorbent

## 1 Data description

**Fig. 1:**
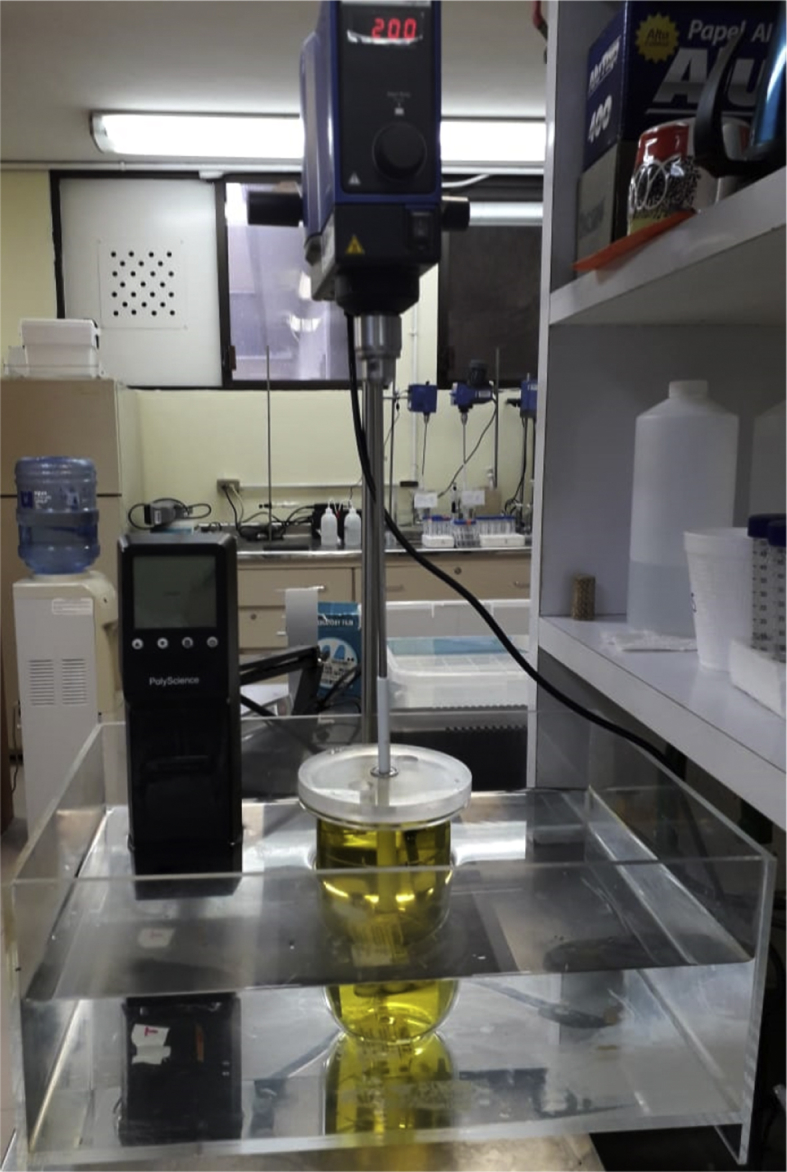
Three-layer glass reactor placed in a constant-temperature bath at 25 °C. The reactor was stirred at 200 rpm with a turbine propeller operated by a rotor.

**Fig. 2:**
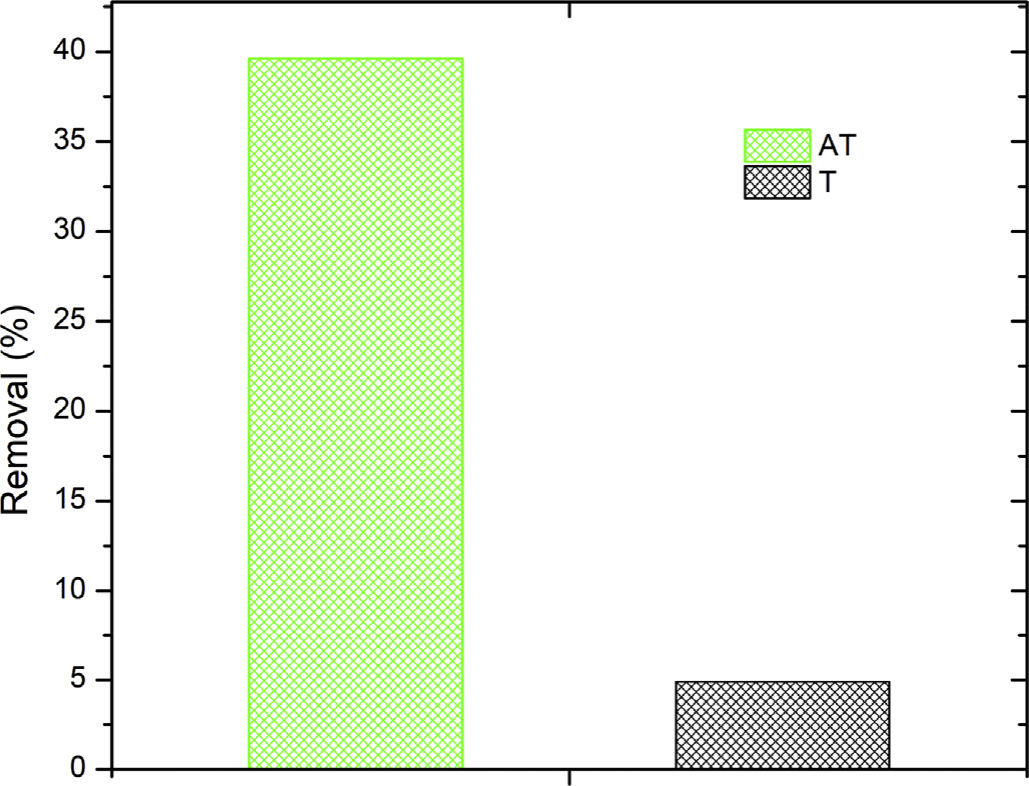
Adsorption of Cr (VI) using materials before and after the transformation process. T: teak wood sawdust; AT: activated teak (solution volume 100 mL, pH 2, Cr (VI) concentration 100 mg L^−1^, adsorbent dose 0.1 g, temperature 25 °C, stirring speed 100 rpm and contact time 3 h).

**Fig. 3:**
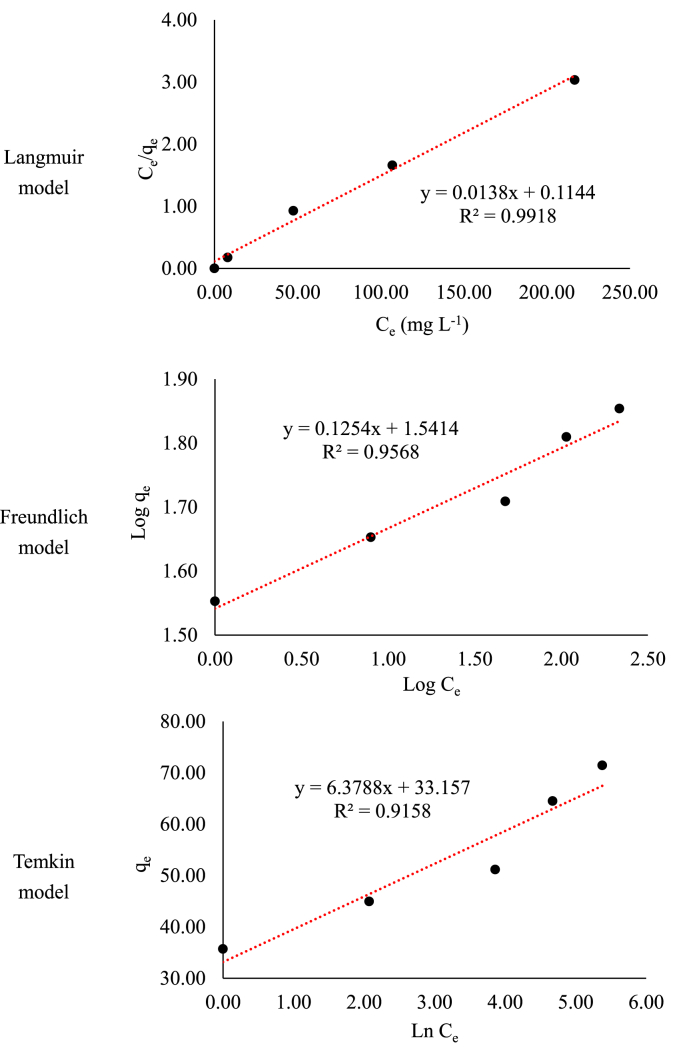
Cr (VI) adsorption isotherms on AT (solution volume 500 mL, adsorbent dose 0.5 g, Cr (VI) concentrations 35, 50, 100, 170, 290 mg L^−1^, pH 2, temperature 25 °C, stirring speed 200 rpm, and contact time 72 h).

**Fig. 4:**
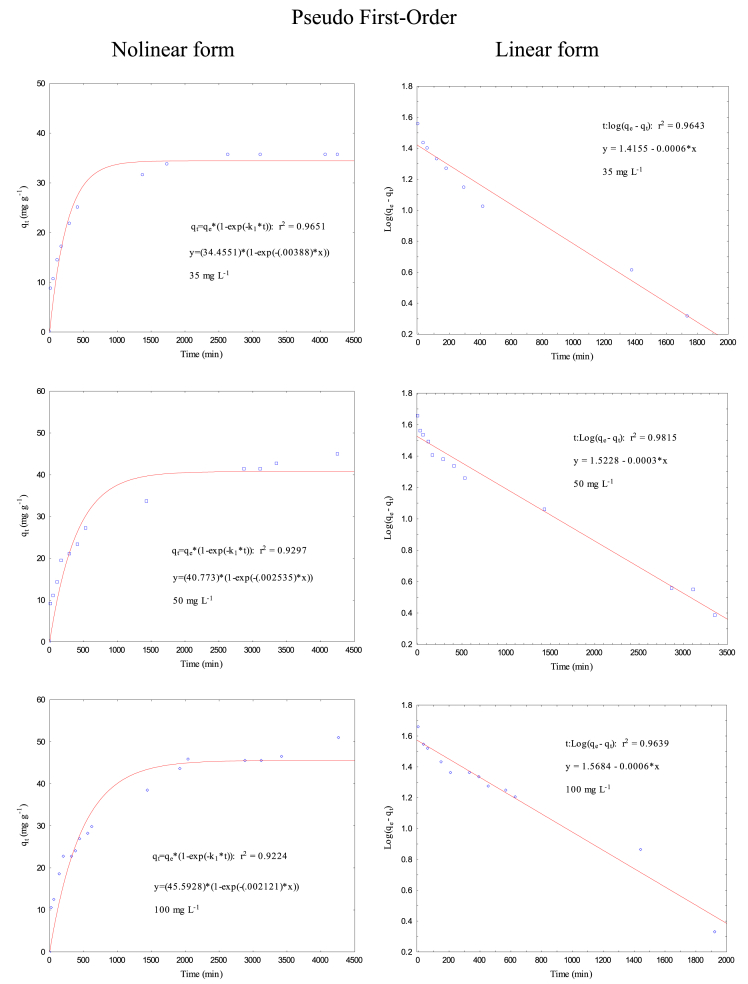

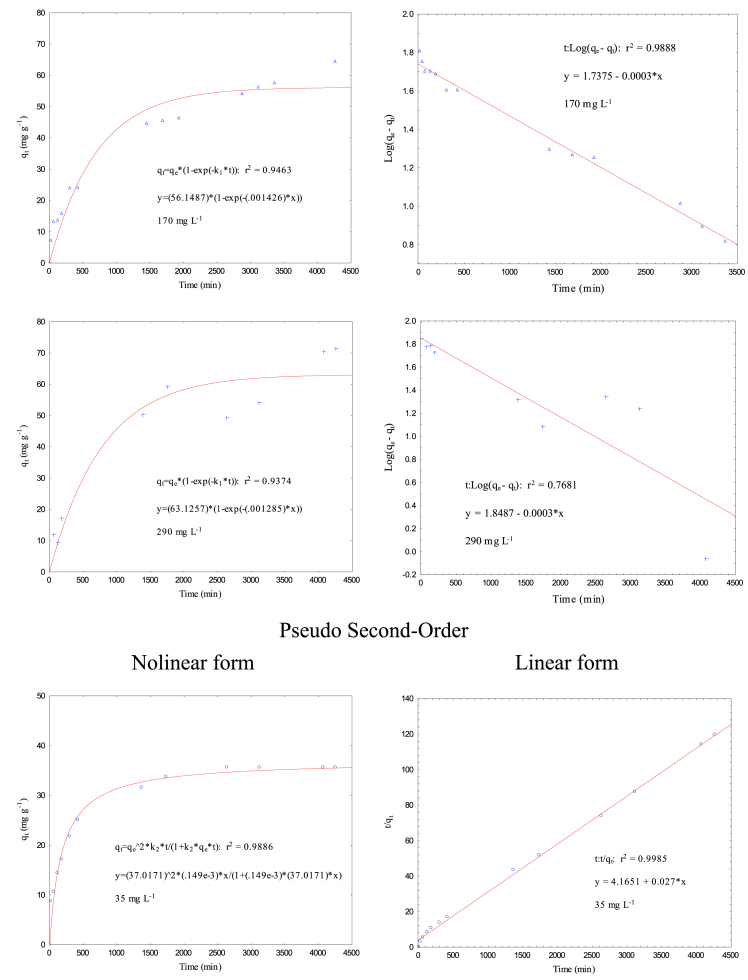

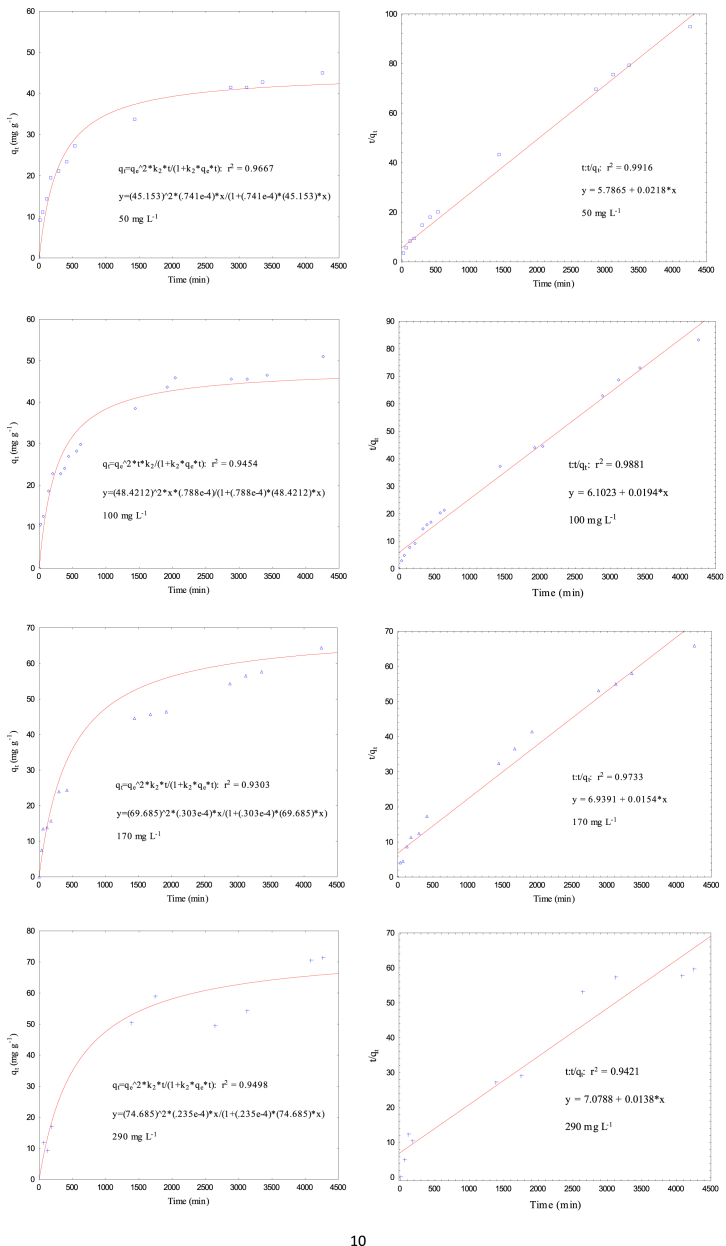
Cr (VI) adsorption kinetics on AT to Pseudo First-Order and Pseudo Second-Order models (solution volume 500 mL, Cr (VI) concentrations 35, 50, 100, 170, 290 mg L^−1^, pH 2, adsorbent dose 0.5 g, temperature 25 °C, stirring speed 200 rpm, and contact time 30, 60, 120, 180, 300, 420, 1380, 1740, 2640, 3120, 4080, and 4320 min). Nolinear and linear form.

Data present in this work correspond to the kinetics adsorption process of Cr(VI) on activated carbon obtained from chemical activation (ZnCl_2_) of teakwood sawdust [1]. Fig. 1 shows the experimental setup where all adsorption experiments were carried out. Fig. 2 shows a comparison between the removal percentage of Cr(VI) using activated and no-activated teakwood sawdust. Fig. 3 presents the data adjustment for the Langmuir [2], Freundlich [3] and Temkin [4] isothermal models. Table 1 and Fig. 4 show the parameters found by nonlinear and linear forms from the Pseudo-first [5] and Pseudo-second order kinetic models [6]. Finally, Table 2 shows the Cartesian coordinates for the most stable configurations during the adsorption process of Cr^3+^ and HCrO_4_^−^ on carbonaceous structures obtaining by Gaussian 09 program. The raw data of all Figures are shared as supplementary material.

**Table 1:** Parameters of pseudo-first order and pseudo second-order models.

Form	C_A0_ (mg L^−1^)	q_e exp_ (mg g^−1^)	Pseudo First-Order			Pseudo Second-Order		
			k_1_ (min^−1^) x 10^−2^	q_e_ (mg g^−1^)	R^2^	k_2_ (g mg^−1^ min^−1^) x 10^−2^	q_e_ (mg g^−1^)	R^2^
Nonlinear	35	35.701	0.388	34.455	0.9651	0.015	37.017	0.9886
Linear			0.138	26.032	0.9643	0.018	37.037	0.9985
Nonlinear	50	41.428	0.254	40.773	0.9297	0.008	44.276	0.9667
Linear			0.069	33.327	0.9815	0.008	45.872	0.9916
Nonlinear	100	45.866	0.212	45.593	0.9224	0.008	48.421	0.9454
Linear			0.138	37.017	0.9639	0.006	51.546	0.9881
Nonlinear	170	64.498	0.143	56.149	0.9463	0.003	69.685	0.9303
Linear			0.069	54.639	0.9888	0.003	64.935	0.9733
Nonlinear	290	71.444	0.129	63.126	0.9374	0.002	74.685	0.9498
Linear			0.069	70.583	0.7681	0.003	72.464	0.9521

**Table 2:** Structural details for the optimization of the most stable configurations for Cr3+ and $HCrO_{4}^{-}$.

^5^Carboxyl-Cr(III)				^7^Phenol-Cr(III)			
6	−4.187693	−0.366274	0.040499	6	−1.030580	3.477402	−0.028900
6	−3.059319	−1.238726	0.049901	6	0.128757	2.686975	−0.009306
6	−1.751557	−0.674547	0.021925	6	0.002722	1.256597	−0.000774
6	−1.577438	0.726102	−0.011577	6	−1.275464	0.655361	−0.006253
6	−2.733738	1.578626	−0.020440	6	−2.443289	1.468870	−0.015426
6	−4.025747	1.001473	0.005383	6	−2.298036	2.882737	−0.030994
6	−0.591101	−1.532645	0.020637	6	1.155929	0.432052	0.012860
6	−0.273356	1.283455	−0.023300	6	−1.381780	−0.754967	0.003851
6	0.889799	0.435463	−0.009962	6	−0.195837	−1.563772	0.001483
6	0.703552	−0.971357	−0.014488	6	1.105480	−0.984962	0.000564
6	2.174201	1.085489	0.019381	6	−0.434359	−2.946158	0.008304
6	2.278795	2.473631	0.002306	6	−1.650731	−3.600612	0.024526
6	1.136990	3.279379	−0.039871	6	−2.787180	−2.795324	0.027557
6	−0.136811	2.710488	−0.045000	6	−2.680645	−1.370234	0.014090
6	−1.308493	3.543158	−0.064102	6	−3.826430	−0.550446	0.009945
6	−2.558343	2.998116	−0.052208	6	−3.715767	0.843030	−0.007004
1	−3.437526	3.633266	−0.063779	1	−4.612741	1.453164	−0.012217
1	−1.174260	4.619354	−0.084685	1	−4.809917	−1.007765	0.018463
1	−5.183318	−0.797106	0.060683	1	−0.945230	4.558680	−0.040533
1	−4.893325	1.652319	−0.001042	1	−3.183777	3.508804	−0.043615
1	3.255824	2.938739	0.025162	1	−1.727405	−4.682396	0.034859
1	1.246454	4.358287	−0.058186	1	−3.773821	−3.249378	0.039726
6	−3.226744	−2.638844	0.084380	6	1.442032	3.260136	0.004054
6	−2.116819	−3.507648	0.091449	6	2.576333	2.477891	0.031647
6	−0.894664	−2.909957	0.057851	6	2.428214	1.078348	0.039128
1	−4.229697	−3.055530	0.105010	1	1.540335	4.340557	−0.004891
1	−2.245023	−4.584250	0.120115	1	3.563101	2.927080	0.047258
6	3.420958	0.329044	0.100924	8	.473129	0.236723	0.076475
1	5.304314	0.432081	0.255699	1	4.350625	0.660436	0.083539
8	4.522211	1.016636	0.213333	24	2.962400	−1.921688	−0.050438
8	3.508512	−0.932318	0.084912				
24	2.255664	−2.312428	−0.167794				

^5^Lactone-Cr(III)				^6^Semiquinone-Cr(III)			
6	−0.832776	3.504155	0.030895	6	−3.833246	−0.355678	0.000010
6	0.303668	2.615118	−0.005207	6	−2.738487	−1.242609	0.000008
6	0.056159	1.206111	−0.012649	6	−1.408411	−0.707168	0.000010
6	−1.238811	0.674113	0.002909	6	−1.226408	0.695654	0.000016
6	−2.354110	1.573249	0.031796	6	−2.341022	1.579887	0.000018
6	−2.100766	2.989299	0.050819	6	−3.644028	1.034067	0.000015
6	1.147262	0.323950	−0.032307	6	−0.272608	−1.589592	0.000003
6	−1.411384	−0.734125	−0.013970	6	0.078159	1.226132	0.000016
6	−0.265677	−1.631638	−0.028680	6	1.168099	0.344252	0.000004
6	1.019198	−1.063121	−0.027080	6	1.037667	−1.054151	−0.000002
6	−0.585170	−2.998919	−0.045517	6	2.513016	0.853897	−0.000017
6	−1.832904	−3.553477	−0.053630	6	2.753805	2.257885	0.000008
6	−2.924352	−2.661115	−0.034092	6	1.673820	3.116087	0.000024
6	−2.730857	−1.262966	−0.011287	6	0.313663	2.646917	0.000020
6	−3.833508	−0.350581	0.016895	6	−0.797106	3.506741	0.000024
6	−3.655416	1.019409	0.040690	6	−2.095721	2.987215	0.000022
1	−4.516540	1.678002	0.064449	1	−2.941803	3.666478	0.000025
1	−4.838897	−0.759476	0.020754	1	−0.650946	4.581176	0.000030
1	−0.670292	4.576210	0.043448	1	−4.841677	−0.755422	0.000007
1	−2.952188	3.660513	0.080105	1	−4.505484	1.693046	0.000016
1	−1.993908	−4.626049	−0.073919	1	3.772272	2.627531	0.000008
1	−3.934731	−3.059489	−0.036702	1	1.845967	4.187561	0.000033
6	1.615468	3.024802	−0.033484	6	−2.929785	−2.659475	0.000003
6	2.466010	0.835923	−0.055411	6	−1.842578	−3.535128	0.000000
1	1.960223	4.050641	−0.036285	6	−0.591631	−2.959496	−0.000001
8	2.665972	2.156415	−0.061853	1	−3.940990	−3.055245	0.000004
8	3.467721	0.060663	−0.072779	1	−1.986370	−4.610063	−0.000003
24	2.951024	−1.846588	0.087127	8	3.461646	−0.037966	−0.000025
				24	2.943861	−1.870998	−0.000041

^4^Carboxyl-$HCrO_{4}^{-}$ - a				^2^Carboxyl-$HCrO_{4}^{-}$ - b			
6	6.246088	0.896100	0.018593	6	−1.406542	−3.987342	0.004927
6	5.054706	1.689010	0.010826	6	−0.401754	−2.972847	0.009852
6	3.798114	1.017034	0.002831	6	−0.803927	−1.607646	0.007171
6	3.728490	−0.406894	0.002519	6	−2.191153	−1.265328	0.000214
6	4.937419	−1.164833	0.010321	6	−3.171358	−2.304556	−0.004853
6	6.186110	−0.475713	0.018325	6	−2.741986	−3.664284	−0.002321
6	2.567345	1.786539	−0.005083	6	0.185608	−0.561586	0.010954
6	2.464715	−1.077938	−0.005394	6	−2.607874	0.103059	−0.001929
6	1.234822	−0.316613	−0.013056	6	−1.618933	1.159949	0.003133
6	1.368670	1.071671	−0.012524	6	−0.288560	0.749278	0.008796
6	−0.010876	−1.014451	−0.020489	6	−2.063594	2.521408	0.001878
6	−0.020541	−2.419826	−0.020685	6	−3.444567	2.802263	−0.004863
6	1.158673	−3.150271	−0.013494	6	−4.388304	1.789664	−0.010364
6	2.419652	−2.501430	−0.005583	6	−3.996110	0.424414	−0.008949
6	3.647794	−3.233378	0.002266	6	−4.951028	−0.637032	−0.014314
6	4.859318	−2.588113	0.009947	6	−4.551308	−1.951308	−0.012368
1	5.782139	−3.161371	0.015958	1	−5.292327	−2.746128	−0.016561
1	3.605371	−4.318798	0.002149	1	−6.008005	−0.386006	−0.019999
1	7.207915	1.400856	0.024817	1	−1.096169	−5.028891	0.006821
1	7.102173	−1.059755	0.024371	1	−3.494456	−4.447972	−0.006294
1	−0.973045	−2.936312	−0.026588	1	−3.769414	3.836122	−0.005800
1	1.123708	−4.235542	−0.013780	1	−5.446249	2.034664	−0.015722
6	5.093516	3.108231	0.010966	6	0.985977	−3.267957	0.016994
6	3.912496	3.863995	0.003314	6	1.947940	−2.267578	0.020295
6	2.718546	3.175872	−0.004269	6	1.578736	−0.906061	0.016233
1	6.055845	3.612815	0.017163	1	1.299080	−4.308754	0.019386
1	3.952253	4.949256	0.003470	1	3.004209	−2.510667	0.022678
6	−1.273716	−0.241201	−0.027577	6	−1.073237	3.604075	0.007856
1	−3.198298	−0.439564	−0.039735	1	−0.891643	5.487864	0.011194
8	−2.374693	−1.005786	−0.034851	8	−1.624212	4.848783	0.007000
8	−1.322941	0.988132	−0.027035	8	0.145855	3.457378	0.013363
24	−6.313063	0.245785	0.024506	24	4.296036	0.372793	−0.003212
8	−4.694395	0.294281	−0.050274	8	2.454133	0.092956	0.018142
8	−6.856321	1.939222	−0.264256	8	4.914575	−1.340584	−0.087988
8	−6.813533	−0.239032	1.465528	8	4.749683	1.037288	1.388596
8	−6.922332	−0.710408	−1.105525	8	4.699979	1.148570	−1.351602
1	−7.818882	2.051534	−0.235191	1	5.881854	−1.395885	−0.108746

^2^Phenol-$HCrO_{4}^{-}$ - a				^2^Phenol-$HCrO_{4}^{-}$ - b			
6	−1.614874	3.160162	−0.003539	6	4.619243	0.062456	0.029576
6	−0.833284	1.970103	−0.027451	6	3.572919	−0.909469	0.013987
6	−1.470031	0.700764	−0.022941	6	2.209210	−0.493167	−0.002249
6	−2.894956	0.652523	0.005241	6	1.918221	0.912454	0.000760
6	−3.662135	1.853858	0.028175	6	2.979178	1.854495	0.017774
6	−2.989383	3.106857	0.023300	6	4.333222	1.400222	0.030977
6	−0.713432	−0.531989	−0.046233	6	1.150974	−1.459785	−0.020229
6	−3.552415	−0.609437	0.009913	6	0.557988	1.371961	−0.009823
6	−2.751780	−1.811296	−0.013019	6	−0.533598	0.452202	−0.027828
6	−1.307109	−1.827091	−0.041221	6	−0.259037	−1.006077	−0.044819
6	−3.499880	−2.987582	−0.006559	6	−1.872245	0.900007	−0.029884
6	−4.873054	−3.120418	0.017898	6	−2.089482	2.301813	−0.018579
6	−5.616389	−1.933307	0.039915	6	−1.044483	3.210566	−0.006846
6	−4.977717	−0.673259	0.036663	6	0.297617	2.773923	−0.001262
6	−5.725105	0.551164	0.059380	6	1.389432	3.698310	0.014516
6	−5.091216	1.764500	0.055524	6	2.682319	3.254835	0.023016
1	−5.669333	2.683885	0.073222	1	3.508247	3.960529	0.035104
1	−6.810204	0.500256	0.079820	1	1.170870	4.762730	0.019903
1	−1.105350	4.119932	−0.007065	1	5.649292	−0.283158	0.040659
1	−3.575146	4.021258	0.041123	1	5.131505	2.136731	0.043180
1	−5.367336	−4.087113	0.020294	1	−3.107754	2.675325	−0.015564
1	−6.702654	−1.978656	0.059654	1	−1.250236	4.278688	0.000410
6	0.587069	2.011333	−0.055900	6	3.863727	−2.293944	0.014533
6	1.347639	0.864164	−0.079587	6	2.854921	−3.229012	0.000464
6	0.721461	−0.410362	−0.075964	6	1.499081	−2.827094	−0.016966
1	1.082582	2.978973	−0.058720	1	4.900484	−2.617943	0.027062
1	2.431945	0.927767	−0.098514	1	3.089391	−4.290299	0.002107
8	1.465945	−1.510551	−0.101349	8	0.536696	−3.783039	−0.029233
1	2.446166	−1.310754	−0.121907	1	0.956579	−4.656752	−0.019870
24	5.314699	−0.194865	−0.042447	24	−3.539618	−0.300913	−0.082132
8	4.082324	−1.248419	−0.185694	8	−1.208090	−1.802789	−0.080415
8	6.372328	−0.422293	−1.219336	8	−4.658388	0.825919	0.154095
8	6.130892	−0.417093	1.545304	8	−3.497070	−1.315233	1.465683
8	4.768133	1.306612	−0.037038	8	−3.611580	−1.086213	−1.492550
1	6.659113	−1.228992	1.594148	1	−4.365320	−1.301285	1.894845
^2^Lactone-$HCrO_{4}^{-}$				^1^Semiquinone-$HCrO_{4}^{-}$			
6	4.019971	−1.822236	0.278019	6	3.069738	−2.930147	−0.000026
6	2.627360	−2.113188	0.071630	6	1.656542	−2.705985	−0.000009
6	1.689127	−1.032886	−0.068373	6	1.192794	−1.360496	−0.000029
6	2.168462	0.302128	0.002643	6	2.086847	−0.261758	−0.000053
6	3.559186	0.570201	0.206870	6	3.496637	−0.517853	−0.000054
6	4.462688	−0.529040	0.341805	6	3.950900	−1.878480	−0.000047
6	0.298348	−1.311860	−0.270774	6	−0.197669	−1.091664	−0.000011
6	1.249181	1.382210	−0.135567	6	1.565296	1.068650	−0.000060
6	−0.143031	1.110689	−0.341863	6	0.122550	1.314607	−0.000069
6	−0.628351	−0.247346	−0.405742	6	−0.736855	0.209804	−0.000023
6	−0.968162	2.223824	−0.486023	6	−0.341676	2.733749	−0.000167
6	−0.571327	3.541767	−0.437562	6	0.685833	3.772654	0.000094
6	0.790818	3.798183	−0.228578	6	2.014101	3.492484	0.000087
6	1.713830	2.736743	−0.077279	6	2.499905	2.139915	−0.000026
6	3.110856	2.967098	0.130891	6	3.888000	1.872639	−0.000022
6	3.994784	1.921319	0.267476	6	4.383997	0.580655	−0.000048
1	5.051444	2.121040	0.423806	1	5.455728	0.403527	−0.000039
1	3.467105	3.992252	0.178123	1	4.575525	2.714742	0.000013
1	4.714474	−2.650549	0.382657	1	3.439726	−3.952196	−0.000014
1	5.517427	−0.320681	0.497966	1	5.021573	−2.063558	−0.000051
1	−1.276286	4.359949	−0.554875	1	0.325204	4.797067	0.000242
1	1.147821	4.823784	−0.183221	1	2.743653	4.299581	0.000197
6	2.154386	−3.404452	0.004311	6	0.692301	−3.743647	0.000026
6	−0.146460	−2.705393	−0.331830	6	−0.675895	−3.487966	0.000044
1	2.756268	−4.299282	0.094547	6	−1.130797	−2.152118	0.000021
8	0.837406	−3.681705	−0.187645	1	1.036917	−4.774942	0.000041
8	−1.276864	−3.132847	−0.494276	1	−1.395153	−4.300543	0.000069
24	−3.509586	0.052897	0.153121	8	−1.542700	3.068030	0.000342
8	−1.905893	−0.461691	−0.644398	8	−2.405667	−1.816222	0.000014
8	−2.809431	0.228089	1.829047	24	−2.903110	0.082466	−0.000009
1	−3.468151	0.476635	2.494917	8	−4.535935	−0.829838	−0.000075
8	−4.539507	−1.179433	0.117018	1	−5.230927	−0.151982	−0.000403
8	−4.051965	1.465179	−0.385886	8	−2.984891	0.958924	1.308051
				8	−2.984919	0.958965	−1.308033

## 2 Experimental design, materials, and methods

### 2.1 Adsorbent material and chromium solution

Adsorbent material was obtained from teakwood sawdust activated with ZnCl_2_ 3 mol L^−1^ (98% CAS 7646-85-7, Duksan) at 550 °C [7]. By adding a K_2_Cr_2_O_7_ (99% CAS 7778-50-9, Mol Labs) mass of 14.145 g to a 1 L volume, a 5000 mg L^−1^ Cr (VI) solution was prepared, from which synthetic Cr (VI) solutions were produced at different initial concentrations (35, 50, 100, 170, 250, and 290 mg L^−1^). The pH level of the solution was adjusted to 2 by using 0.1 mol L^−1^ NaOH (98% CAS 1310-73-2, Panreac) and 0.1 mol L^−1^ HCl solutions (37% CAS 7647-01-0, Merck).

### 2.2 Reactive system

Fig. 1 shows the reaction systems used during the adsorption experiments of Cr (VI) on activated teak (AT). 500 mL of each solution, at pH 2 and 0.5 g of AT, was brought in contact with a three-layer glass reactor placed in a constant-temperature bath at 25 °C. The reactor was stirred at 200 rpm with a turbine propeller operated by a rotor. Samples were taken at 30, 60, 120, 180, 300, 420, 1380, 1740, 2640, 3120, 4080, and 4320 min, until reaching equilibrium. The Cr (VI) concentration of each sample was measured using a Shimadzu UV 1900 UV/VIS spectrophotometer at 542-nm wavelength.

### 2.3 Experimental adsorption

Fig. 2 shows that the activation process of teakwood sawdust with ZnCl_2_ improves the adsorption of Cr (VI). The removal rate increased for AT 8 times respect to T.

Fig. 3 shows the linear fit of the experimental data to the Langmuir, Freundlich and Temkin isotherm models. Equilibrium experiments were carried out using Cr (VI) solutions at different initial concentrations (35, 50, 100, 170, 250 and 290 mg L^−1^), adsorbent dose of 0.5 g, temperature of 25 °C, stirring speed of 200 rpm and adjusted the solution pH at 2 with an optimal contact time of 4500 min. The experimental data were treated mathematically using the Excel 2013 software to calculate the isotherm parameters, as follows: when plotting C_e_/*q*_*e*_ based on C_e_, *K*_*L*_ and *q*, two parameters (K_L_ and q_max_) can be obtained by using the slope and the intercept (Langmuir constants). When plotting *Log q*_*e*_ against *Log* C_e_, *K*_*F*_ and *1/n* (Freundlich constants) are estimated. By plotting *q*_*e*_ against *Ln C*_*e*_, *K*_*T*_ and *b* (Temkin constants) are calculated.

Table 1 y Fig. 4 present the obtained parameters from the experimental data adjusted to nolineal and lineal kinetics models (Pseudo-first and Pseudo second order). The experimental data were treated mathematically using the Excel 2013 software. Constants from lineal form were calculated as follows: for the PFO model, a *Log (q*_*e*_*-q*_*t*_) graph was developed as a function of time (*t)*, from which the values of *q*_*e*_ and *k*_*1*_ were calculated. In addition, for the PSO model, *q*_*e*_ and *k*_*2*_ were calculated by plotting *t/q*_*t*_ against *t*. To calculate nonlinear shape constants of the kinetic models, the least squares model derived from the Rosenbrock–Newton optimization algorithm was applied through the Statistical software.

*C*_*A0*_ (mg L^−1^) is the initial Cr (VI) concentration of the solution; *k*_*1*_ (min^−1^) is the pseudo first-order rate constant; *q*_*e*_ is the amounts of Cr (VI) adsorbed in the equilibrium (mg g^−1^); *k*_*2*_ (g mg^−1^ min^−1^) is the pseudo second-order rate constant; *R*^*2*^ is the correlation coefficient.

Table 2 shows the Cartesian coordinates for the most stable configurations during the adsorption process of Cr^3+^ and HCrO_4_^−^ on carbonaceous structures.
